# A parent-led, patient-centered medical home model instruction for interprofessional undergraduate and graduate learning opportunities

**DOI:** 10.1080/10872981.2021.2012105

**Published:** 2021-12-17

**Authors:** Lori Heginbotham, Gina Baugh, Timothy Lefeber, Linda Friehling, Christy Barnhart, Lee Ann Miller, Lucas Moore, Lesley Cottrell

**Affiliations:** a Center for Excellence in Disabilities, West Virginia University, Morgantown, WV, USA; b Director of the Wv Office of Interprofessional Education, West Virginia University School of Pharmacy, Morgantown, WV, USA; c Department of Pediatrics, West Virginia University, Morgantown, WV, USA; d Nursing Workshop, West Virginia University Adult Health Department; e West Virginia University, Patient Safety Simulation Center (WV STEPS), Morgantown, WV, USA

**Keywords:** Team-based learning, patient-centered medical home model, chronic illness education, family support services, parent-provider communication

## Abstract

**Introduction:**

Despite national efforts to establish patient-centered medical homes (PCMH), 57.3% of children with special health care needs are receiving care that does not meet medical home criteria. Project DOCC, a national curriculum designed by parents of children with disabilities or chronic disease, has shown documented strengths in medical resident learner education of children with special health care needs from the parent perspectives for over a decade. Because of the importance of PCMH and the need to provide compassionate care, our team adapted the curriculum to incorporate team-based learning in the rural setting.

**Materials and Methods:**

Reading materials were distributed to learners prior to an in-person workshop at which time, learners reviewed a video and discussed PCMH materials to identify elements of the PCMH. Learners then engaged with parent mentors across three breakout sessions. A final group reflection was completed to review and discuss efforts providers would take to establish and maintain the PCMH in their own practice. Baseline and post-workshop PCMH perceptions and parent mentor reflections were collected and compared using t-test comparisons.

**Results:**

Learner knowledge, perceptions, and comfort significantly increased after the workshop. Parent mentor comments also highlighted an increased understanding for the provider. **Discussion:** The adapted PCMH curriculum significantly impacted learner outcomes using a feasible approach that fit nicely within health professional curricula and limited resources of the rural setting. Parents enjoyed the opportunity to serve as mentors and valued the instruction format.

## Introduction

The patient-centered medical home (PCMH) model aims to improve health care for patients and their families by addressing the quality of the healthcare experience while lowering the overall costs. [1,2] The PCMH is more than a theoretical concept and has been supported in more than 498 articles since 2000 [3]. The growing volume of work on the PCMH has consistently described an effective PCMH as one that: 1) provides comprehensive care that is effective for the patient’s needs and tailored to available resources and challenges; 2) is patient-centered, which means focused on all aspects of the individual including his/her larger culture and value system; 3) provides coordinated services to avoid duplications; 4) provides accessible services to ensure all patients have access to the care they need; and 5) is committed to quality improvement using an evidence-based approach [1]. Asarnow et al [4]. demonstrated that greater engagement from a patient and family in medical home team discussions has had a documented positive effect on family knowledge, self-efficacy, and select health outcomes including service utilization and costs [5–7]. The PCMH premise is that it is necessary to include the patient and/or family representative(s) in the healthcare decision-making and implementation processes to improve healthcare for patients particularly those with complex medical needs.

Despite being an evidence-based model, the PCMH can be a challenge to implement [3,4]. Initiating and sustaining a PCMH can be costly to change systems and procedures and taxing on provider time. The process of establishing a medical home can also be complex and involve input from others beyond the immediate providers on-site. Input from all members of the medical model are key especially for translating the recommended treatment beyond the clinical setting and into the community and home. Interventions can be complex and require additional provider or clinical staff coordination among themselves and across disciplines. [8,9] Thus, in order for the PCMH to be successfully implemented, providers must have the knowledge and tools they need to invite the patient and/or family representatives into the medical home process, support partner contribution to the process, and sustain a collaborative model over time.

Early PCMH studies largely attempted to change the clinical infrastructure (e.g., payor source) to see an effect on PCMH outcomes. [9, 10–13] However, in 2019, Leung and colleagues reviewed 54 manuscripts and documents for any efforts made outside of the clinical structure that would enhance, and sustain, a collaborative team in the decision-making model. They conducted focus groups with patients and family members about their experiences with the medical home and their healthcare process in general. Their findings highlighted organizational efforts to involve the patient and/or a family representative more often to develop personal health care treatment plans. They documented organizations that would also provide patients and families interactive tools designed to give them relevant information about their health and an ability to reach out to providers with questions or suggestions (e.g., patient portal). Leung and colleagues emphasized a shift in how organizations were viewing PCMH noting the importance of patient and family involvement in the process. The studies reviewed, as well as others that followed, emphasized the importance of identifying a clinical champion who believes the PCMH is necessary for patient success. The authors urged future clinicians to identify best practices for involving patients and families siting the need to provide clear expectations, roles, and responsibilities for all stakeholders, prioritize effective communication with regular updates, engage in groups of three or more so they can encourage each other and benefit from shared discussions, and providing increased time to practice these skills. They argued that while these skills are challenging for the most experienced provider, it is important for clinical teams to have this awareness and these skills early in their career.

Curriculum-based efforts for addressing the PCMH training gaps are limited but largely effective. Curricular offerings for undergraduate and professional learners have largely focused on identifying and describing PCMH elements to increase PCMH knowledge and awareness [14–16]. Previous evaluations of PCMH curricula also largely focused on the learner’s experience with the interprofessional training process (e.g., warm hand off to another profession; identifying own discipline’s role in process, and benefits of teamwork in the process) [17–21]. Very few training efforts have directly involved the patient or family in the development or implementation efforts of a training opportunity [22]. Sheppard and colleagues provided a curriculum to medical students using a flipped classroom approach to review special education law and practices [23]. Parents of children with special health care needs engaged with students through a panel discussion. These collective efforts significantly increased student understanding of special education laws and familiarity engaging with the parents. Similarly, Parent and colleagues implemented a longitudinal curriculum including home visits and didactic presentations focusing on parents as mentors and their family stories [24]. This initiative was also effective in increasing learner knowledge and skills engaging with parents of special healthcare needs. Parents and/or family representatives; however, had not contributed to the development or contributed to the implementation of a curriculum in many instances.

In 1994, parents of children with special health care needs uniquely contributed and led a curriculum (Project Delivery of Chronic Care (DOCC)) for pediatric residents focused on parent experiences with the healthcare system [25]. The five-hour curriculum included a grand rounds panel discussion, home visit, and parent interview to describe their experiences with the medical care model, decision-making process, and transition from the medical setting to home and community. This approach has since been applied to disciplines beyond pediatrics including psychiatry, oncology, and family medicine and has been a consistently effective approach for illustrating and injecting the key experiences of the patient and family into the discussion [26,27]. While effective, this particular implementation approach may not always be feasible.

In this study, we considered the rural setting, family challenges and resources for participating as active mentors, and the infrastructure and schedule of our health sciences curricula. To be considered as a curricular option, the Project DOCC curriculum needed to be adapted. Therefore, we not only tested a potentially effective way to incorporate the parent/family into the medical home discussions and process but explored the feasibility of this training being implemented across multiple disciplines so that knowledge about the PCMH and skills for initiating the parent/family involvement are available to the team. The objective of this study was therefore, to describe the final content and organization of an interprofessional team workshop, test the perceived effectiveness of the workshop in terms of the PCMH awareness and skills, and describe experiences from the student and parent perspectives.

## Materials and methods

### Curriculum adaptation

Our team initially met with the Project DOCC developers, family members, faculty mentors, and an initial group of learners to discuss how the original format would need to be modified for our learner schedules and based on our resources. These discussions highlighted the need to develop materials that: 1) represent pivotal positions of participating disciplines on the PCMH; 2) could be implemented within the curriculum (undergraduate and graduate as well as across health sciences disciplines) at various points; and 3) could be implemented with minimal resources or preparatory planning. The following activities were originally developed following that meeting and have been piloted among three cohorts of learners.

#### Pre-workshop activity

Select documents were available on-line through our health science educational portal, SOLE. The SOLE platform is secure and accessible by all health sciences learners, faculty, and staff. Three documents were shared two weeks prior to the scheduled simulation activity that included:

- A policy statement from the American Academy of Pediatrics entitled, ‘Patient- and Family-Centered Care and the Pediatrician’s Role’ [28] (Appendix B)
- A reference to People First Language (Appendix C)
- Questions planned for the team-based activity (Appendix D)

Learners were able to send faculty mentors any questions they had about the materials and completed the baseline assessment on-line. Family members who were serving as mentors in the training could also review their roles and guidelines prior to the workshop.

The Project DOCC module within SOLE became available in each learner’s platform at this time and stayed open through the duration of the simulation. The purpose of this segment was to provide essential policy statements on PCMH, introductory guidelines for using person first language, and an overview of the purpose of the simulation and procedures. Once reviewed, learners also obtained the baseline assessment items to complete prior to the simulation activity.

#### Parent mentor training

Parents who agreed to serve as parent mentors received a one-hour training by phone or conferencing software. Parent mentors also received a parent mentor preparation materials and overview document (Appendix E) including the introduction index card and soap-box card (Appendix F) to have ready for the in-person workshop and discussion. The packet also included sample parent mentor interview questions that learners may cover during the in-person workshop (Appendix G).

Specifically, this training reviewed the curriculum and specific points where parent mentors and learners interacted. The training also included a review of the PCMH elements with a question and answer period for the parent mentors at the end of the training. Providing a parent mentor training allowed the team to ensure parent mentors had a similar understanding of the purpose and layout of the simulation activities. It also allowed the team to work out logistics such as timing for each segment and order.

#### Workshop activity large group overview

An in-person workshop was conducted in the WVU Simulation Training & Education for Patient Safety Center (STEPS). All learners, faculty mentors, and family mentors began the workshop together with a large group discussion. This segment lasted for 45 min. Faculty and family mentors initiated the large group portion with a video presentation according to the roles and guidelines they had reviewed prior to the workshop. This time was important for learners and mentors to meet one another, to utilize narrative medicine to orient learners to three representative families and their daily life experiences, to review the importance of the PCMH, particularly for families of children with special health-care needs, and to review the components of PCMH.

The author-owned video content presented medical experiences from the perspective of three families. Each story highlighted family experiences with their children’s health condition, their day-to-day schedules, short descriptions of their experiences with health-care providers, and final points each family thought was important for providers to know when establishing health-care regimens. The group discussed the video immediately following the use of the proposed discussion questions found within the Instructors Guide. Specifically, learners were asked to identify elements of the video they wanted to highlight. The group also discussed how the family stories would be helpful to each discipline: nursing, medicine, and pharmacy noting any similarities and differences.

#### Workshop activity: small group discussions

Following the group discussion, learners were randomly assigned to one of three small groups. Each small group had at least one learner representative from each discipline. Each group was assigned a family mentor with which they would meet for a short group discussion (15–20 minutes) and then transitioned to a different family mentor. They continued this process until they had met with three family mentors (60 minutes total). While the original Project DOCC curriculum provided a list of interview questions titled the ‘Chronic Illness History’, learners in this adaptation were asked to craft their own questions for the family mentors when given the task of assessing the family’s experience with care and the role their future profession played (or failed to play). The purpose of this segment was for learners to identify how the role of their discipline impacts these families and how they as providers may work with a parent to enhance care.

During the small breakout sessions, learners and family mentors followed the team-based activity questions that had been given to them prior to the workshop. This document posed the task of discussing what is a medical home and how should it function, what is patient-centered care, and what barriers do families experience when receiving patient-centered care within a medical home? The second task within each group was to think of at least one question that would access each element of the PCMH: accessible, patient/family-centered, continuous, comprehensive, coordinated, compassionate, and culturally effective. Interactions between learners and a family mentor were observed by faculty and family team members in another room within the simulation center.

#### Workshop activity large group review

Following the small breakout sessions, all learners were brought back into the larger group discussion to conclude the activity. In the last 45 minutes, learners summarized highlights from the various discussions. A structured reporting format was not required. This section of the workshop was established to get impressions and feedback from all stakeholders. Family mentors shared their experiences in the discussions noting learner demeanor, questions, and comfort. Similarly, learners shared their experiences with the activity describing family mentor reactions, additional questions, and their personal feelings before and after the exercise.

Setting

The training program was established within a clinical simulation center in a rural health science center. Simulation center space included a moderate (18–20 persons) lecture hall and three separate clinic rooms. All rooms offered video and audio recordings and media (e.g., computer, projector, internet) to share slides, videos, and other information. An outline of the preparatory, in-person, and follow-up procedures were reviewed and updated by the study team regularly (Appendix A).

Team Formation

Learner teams were formed prior to each implementation by the interprofessional team of faculty mentors. Up to five teams with 3–4 learners were created for this exercise. Each team included learners from undergraduate (senior) Nursing Community Health, third-year Pharmacy, third year undergraduate medicine, and medical resident programs. Group size was determined based on the need for small group discussions representing all participating disciplines.

Immediate Feedback

Immediate feedback was given to learners after they had completed baseline questions that pulled information from the policy statement, people first language document. Learner knowledge about the PCMH following the workshop also incorporated discussions from the initial group discussion within the workshop. Faculty and family mentors were available for questions during the video and group discussion. Family mentors also responded to learners’ questions during the small breakout sessions.

Facilitation Schema

The team-based learning activity required a total of 3.5 hours:

- 1-hour family mentor training
- 45 minutes video review and response
- 60 minutes family scenarios
- 45 minutes closing and evaluation

Evaluation Strategy

Baseline and post-workshop survey items assessed learner PCMH definition and elements that define it, the learner’s familiarity with the PCMH prior to the workshop, perceived contribution of professional role within elements of the PCMH as part of the interprofessional workshop, and experience working with medically complex patients prior to the workshop. Baseline surveys were distributed by email to registered learners one week before the workshop. Post-workshop survey items were distributed by email one hour after the workshop ended. In both instances, surveys were left open for one week to complete. A reminder was sent by email three days after the initial email.

Perceived awareness of the PCMH and experience providing care for patients who are medically complex responses were based on a 5-point modified Likert scale with 1 “not at all aware and 5 ‘completely aware’. Learners’ perceived understanding of their profession’s role in providing care within the PCMH was also based on a 5-point modified Likert with 1 ‘not at all aware’ and 5 ‘completely aware’. Finally, learners were asked how important each individual (i.e., primary care provider (PCP), medical specialists, patient, parent, nurse, pharmacist social worker) would be important when caring for the medically complex pediatric patient based on a 5-point modified Likert scale ranging from 1 ‘not at all important’ to 5 ‘completely needed’.

Three additional questions were administered post-workshop to learners to assess their perceptions of any barriers toward implementing the PCMH and the impact of the workshop and experience overall. Learners were asked to rate three aspects of the learning experience plus an additional item of their choice marked ‘other’ in terms of its impact on their understanding of the medical home model and their approach to children with special health-care needs. Learners could respond using a 4-point modified Likert scale ranging from 1 ‘not at all’ to 5 ‘to a great extent’. The final item assessed learner perceptions of how well they personally accomplished the following: communicate with patient and family; develop a working knowledge of specific medical problems to be followed; show positive and compassionate attitude toward the patients and families; and seek additional encounters and ask questions. Responses were based on a 4-point modified Likert scale ranging from 1 ‘not at all’ to 5 ‘completely’.

Family mentors completed post-workshop surveys responding to questions about how the workshop should be changed in future renditions, did the learners appear to be culturally sensitive, and how the training experience impacted them personally. This post-workshop survey for the family mentor was distributed by email one hour after the workshop ended and remained open for one week. All proposed activities and analyses were reviewed and approved by the West Virginia University Institutional Review Board (IRB – protocol # 1,304,035,754).

We conducted descriptive statistics to describe sample mean distributions of baseline and post-workshop responses. Learner baseline and post-workshop responses were then compared using a dependent sample t-test comparisons. Parent mentor responses were descriptive in nature. Significance level at the p < 0.05 was used. Analyses were conducted using SPSS version 26.0 [29].

## Results

The workshop and preparatory steps were conducted four times for the purposes of this study. Sixty-five learners participated across four cohorts (cohort 1 = 15 learners; 2 = 20; 3 = 15; 4 = 15) during the workshop. Each cohort included at least three learners from each of the following programs: undergraduate (senior) Nursing Community Health, third-year Pharmacy, third year undergraduate medicine, and medical residents in Pediatrics at various stages of training. Cohorts of at least nine trainees were conducted in March, September, and December, 2019. The final layout of the adapted Project DOCC was implemented in January 2020.

### Learner perceptions of PCMH and professional contributions

Table 1 provides mean comparisons for learner outcomes at baseline and post-workshop. The activity significantly improved learner knowledge of PCMH and PCMH elements [Baseline X = 1.68 (SD = 1.2) vs Post X = 4.22 (1.0)]. Learner awareness of their professions role within individual element of the PCMH also improved for: providing continuous care [Baseline X = 2.17 (1.4) vs Post 4.32 (0.8)], family-centered care [Baseline X = 2.34 (1.4) vs Post X 4.49 (0.6)], coordinated care [Baseline X = 2.41 (1.5) vs Post X = 3.98 (1.1)], accessible services [Baseline X = 2.22 (1.4) vs Post X = 4.15 (1.0)], and culturally effective treatment [Baseline X = 2.63 (1.4) vs Post X = 4.15 (1.0)]. Learner perceptions of how important particularly professions were within the PCMH did not significantly change with regard to the PCP, patient, parents, pharmacist, and social Worker [Baseline X = 4.29 (1.1) vs Post X = 4.61 (0.8)]. Learner baseline reports noted great importance for these professions already [Baseline X = 4.10 (1.3) vs Post 4.61 (0.8)]. However, their views of the Medical Specialist’s [Baseline X = 4.10 (1.3) vs Post X = 4.61 (0.8)] and Nurse’s role [Baseline X = 4.26 (1.2) vs Post X = 4.58 (0.8)] significantly increased between baseline and post-activities.

**Table 1.** Mean comparisons of trainee outcomes at baseline and post workshop

Outcome	Mean (SD) Baseline	Mean (SD) Post-Simulation	P value
Familiarity with PCMH	1.68 (1.21)	4.22 (1.03)	<.001
Role of profession in continuous care element	2.17 (1.43)	4.32 (0.84)	<.001
Role of profession in patient, family-centered element	2.34 (1.45)	4.49 (0.62)	<.001
Role of profession in coordinated care element	2.41 (1.52)	4.39 (0.73)	<.001
Role of profession in accessible services element	2.22 (1.43)	3.98 (1.12)	<.001
Role of profession in culturally effective element	2.63 (1.46)	4.15 (1.07)	<.001
Importance of PCP in PCMH	4.29 (1.13)	4.61 (0.82)	NS
Importance of Medical Specialist	4.10 (1.34)	4.61 (0.81)	<.012
Patient	4.35 (1.25)	4.68 (0.71)	NS
Parents	4.45 (1.11)	4.71 (0.65)	NS
Nurses	4.26 (1.26)	4.58 (0.88)	<.05
Pharmacist	4.00 (1.37)	4.32 (1.03)	NS
Social Worker	4.10 (1.33)	4.52 (0.92)	NS

Note. Abbreviations: Standard deviation (SD); Parent Centered Medical Home (PCMH); Primary Care Provider (PCP); p value = significance; t-test comparisons by item provided based on response options ranging from 1 (not at all) to 5 (to a great extent).

### Perceived workshop effects

**Figure 1.**
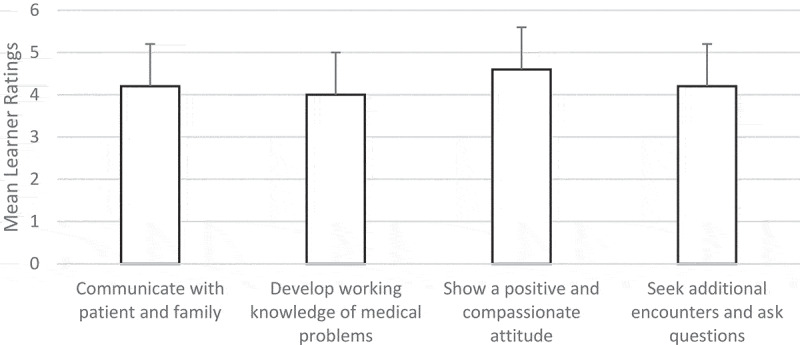
Mean learner reports of simulation effects on select skills

Learners rated certain elements of the activity in terms of its impact on their understanding of the PCMH. The average rating for the background information about the PCMH provided pre-workshop was 3.93 (SD = 1.1). Other elements received average ratings including: the workshop of patient visit (X = 4.56, SD = 1.0) and debriefing of workshop with families (X = 4.41, SD = 1.1). Overall, learners thought the adapted Project Delivery of Chronic Care (DOCC) activities effectively contributed to their knowledge of the PCMH, their ability to communicate with the patient and family to develop effective treatment programs, and collected family perspectives that improved their attitudes and compassion toward patients and families (see Figure 1).

### Parent mentor post workshop thoughts

Family mentors provided additional feedback about the workshop and supportive activities. This information would be used to strengthen future encounters with learners as a quality improvement tool. Family mentors suggested more opportunities prior to the workshop that would encourage learners to ask direct questions of mentors during the workshop. For instance, following this feedback, the team added language to the workshop noting the opportunity to engage with the family in each segment and the opportunity to ask any question while they were in the room. Similarly, they thought mentors would need to ask more direct questions to the learners during the workshop to enhance their involvement in the activity. Prompts from the parents asking about previous experiences with PCMH or families were added to the parent training as a result. Family mentors felt that learners, for the most part, appeared interested in learning more about the family and that those interests increased as the series of activities took place. In terms of the impact the activity had on them personally, many family mentors noted, ‘It confirmed that my expectations as a parent are not unrealistic’. Another family mentor noted, ‘I am pleased to see the impact of the program to help the learners better understand how it is important to have reasonable expectations for the families’. These statements are representative of the typical comments provided by family mentors.

## Discussion

This study established a PCMH training that included parents, faculty, and learners in a series of activities (on-line and in-person) that improved learner knowledge of the PCMH and skills necessary for establishing a PCMH in their future practice. The documented tools and processes increasing learner PCMH knowledge, improving their capacity to engage parents to gather information about their child’s condition and experiences with treatment, and enhanced their comfort with receiving parent feedback and increased involvement. This training intervention is unique from established curricula in that it can be presented to larger groups of interprofessional learners and focuses on the skills needed to establish an effective PCMH.

Learner and family mentor feedback on the experience supported the added value of the interprofessional nature noting how it was helpful to hear how each discipline plays a different but significant role in the process. Each learner would ask families questions from their future profession’s perspective. However, learner questions built upon one another as the discussion with families as mentors continued.

Workshop conversations met similar criteria of those from the home visit or interview in the original curriculum while allowing learners to remain on campus and available for other course obligations. An unexpected finding from family and learner feedback demonstrated that we could provide a valuable interaction and incorporate these interactions into complex schedules. This allowed us to offer the training more often (monthly) and reach as many learners as possible.

## Limitations

This study is cross-sectional and, therefore, unable to describe potential changes in learner perceptions or skills of PCMH over time. Similarly, parent mentor perceptions of the workshop and interaction over time may change for various reasons. The survey used in this study was designed for the specific purpose of assessing learner and parent perceptions of PCMH from the workshop. Assessments of learner PCMH understanding are limited in number and are limited to self-report from learners and parent mentors in this study. Future studies should consider validating measures for assessing these outcomes. Attained learner skills following the workshop should also be captured in future studies.

## Conclusions

In our adaptation of the Project DOCC curriculum, we learned several lessons. First, it is important to identify a faculty champion from each discipline as well as parent mentor champion. This was important not only important for learner recruitment but for understanding the most appropriate times to interject the opportunity within the curriculum for each discipline. We also noted it is important to establish a plan for parent participation that is flexible and allows some fluctuation and support given the complexities in their lives. Future adaptations may consider teleconference so that parents can serve as mentors without traveling long distances or having to schedule significant portions of their time away from their children. The third lesson learned pertained to the amount of time learners and parents needed during the workshop and in the debriefing with the whole group. Throughout the pilot, we identified key concepts to reach for each segment and attempted to complete those within the set period of time. Finally, it is important to consider compensation for parents for their time and travel. This may require internal or external funding but is helpful for the consistency and family availability.

While our adapted workshop was intended to increase knowledge and perceived skills within the context of children with special health-care needs and their care, the activities, discussions, and elements could arguably be adapted for other populations for the same purpose. Future studies would have the opportunity to use the proposed infrastructure, prompts, and questions to assess whether the same effect would be achieved for other patient groups.

## Appendix A Appendices

Appendix A.

**Family Experience/Project DOCC**

(Delivery of Chronic Care)

**Implementation Guide**

*IPE Workshop Experience*

The purpose of this implementation guide is to provide information about the individual components of the Family Experience/Project DOCC program and to review best practices for establishing similar experiences at your institution. These activities have successfully been provided to a broad audience of health science students including undergraduate and graduate medical students, pharmacy, and nursing. Prompts, measures, and communications are included in this guide to get the most benefit from the interprofessional group experience.

**INTRODUCTION**:

There is a growing need for a comprehensive approach to caring for medically complex children in primary care settings since more infants are surviving extreme prematurity, complex congenital heart disease, childhood cancers, and a growing number of genetic diseases. Parents of medically complex children can be stressed and confused by daily demands of coordinating care between specialists, therapies, and community. Although they regularly interact with multiple specialists, parents often feel that no one is ‘driving the bus’ and that their voices are not heard.

**This parent-led workshop training experience for medical residents and health professional students is designed to demonstrate how the patient-centered medical home model functions in the care of medically complex pediatric patients**. The workshop exercise emerged as an adaptation of the 1994 Project DOCC (Delivery of Chronic Care), national curriculum that was designed by parents of children who have chronic illness or disability, and uses parents as the teachers. The current version has been adapted so that it can function successfully as a workshop inter-professional educational experience (IPE) for learners. The exercise consists of four phases: 1) pre-workshop; 2) pre-briefing; 3) workshop/exam room/parent interview; and 4) debriefing session.

**PURPOSE/GOALS/OBJECTIVES**

**Purpose**: The overall purpose of this workshop is to introduce medical residents, medical, nursing, and pharmacy students to the way in which the Patient Centered Medical Home model functions in the care of the medically complex pediatric patient. The goal is two-fold (1) assess the degree to which the family has experienced patient-centered care within a medical home (2) explore the role their future profession played or failed to play in a family’s experience.

**Goals**:

1. Learners will collaborate in an inter-professional team to assess the challenges families with Children with Special Healthcare Needs face, each member of the team taking responsibility for specific defined roles.
2. Learners will be able to list the ways in which a Patient Centered Medical Home model, placing the parent/caregiver in a pivotal teaching role, addresses the challenges of coordinating care for these complex patients.

**Learning Objectives**:

1. Define patient centered medical home care in terms of accessible, continuous, comprehensive, family centered, coordinated, compassionate, and culturally effective
2. Describe elements that designate a pediatric patient as medically complex
3. Describe the role of your respective professional program
4. Identify elements of family-centered care
5. Identify and analyze common barriers to successful implementation of the Patient-Centered Medical Home (PCMH) model

**TRAINEES**

This workshop exercise can be offered as an Inter-professional Education opportunity for: 1) 3^rd^ and/or 4^th^ year medical students; 2) medical residents; 3) pharmacy students; and 4) nursing students.

**PARENT MENTORS**

Criteria

- Potential parent mentor must have a need for coordinated care, meaning that they see multiple specialists, and have both a physical and developmental disability (i.e., a medically fragile child that also has autism, down syndrome, etc.)

- Foster parents are also able to participate (as long as they have cared for the child a reasonable amount of time to understand their chronic care needs)

**PRE-WORKSHOP PREPARATION & TRAINING**

I. **Standardized Patients/Parents of Children/Youth with Special Health-Care Needs (CYSHCN)**

Participating parent mentors are sent a Participation Packet that includes the following:

- Letter from team member
- Workshop Day Instructions (directions, parking, agenda, cancellation contact person)
- Introduction Card and Soap Box Card Templates
- List of sample questions that trainees may ask
- Documents that may be needed (e.g., W-9) if travel is reimbursed
  II. **Standardized Parent Mentor Training**

Participating parents will receive a 60-min training course on-site by parent leaders on the day of the workshop.

III. **Interprofessional Trainee Group Pre-Materials**

The Pre-Assessment is sent to trainees 7–10 days prior through a secure, online learning portal or other electronic system used at your particular institution. Once the Pre-Assessment is completed, the trainee receives the Pre-Materials Packet through the same communication channels.

Pre-Materials Packet includes:

1. Pre-Assessment
2. Goal/Objectives/Approach/Process
3. AAP Policy Statement on Patient- and Family-Centered Care
4. People First Language

**WORKSHOP DAY SCHEDULE**

The second phase of this opportunity includes activities that can take place in-person or virtually. A sample schedule is provided below.

Sample Schedule

**Table:**

Time		Description
TRAINEES	PARENT MENTORS	
	12:30–1:30	Parent Mentor Training Workshop
	1:30–1:45	Parent mentors break and then escorted to assigned separate rooms to meet trainees
1:00–1:45		Trainee **Pre-Briefing** Session
1:45–2:30	1:45–2:30	**Workshop Experience**/Parent Mentor interviews in separate ‘exam rooms’
	2:30–2:45	Parent mentors leave and return to separate room for group reflection with faculty mentors
	2:45–3:00	Parent mentors complete Experience Survey & Feedback on computers
2:30–3:00		Trainees report to **De-Briefing** area for group reflection with parent and faculty mentors
3:00–3:30	3:00–3:30	**Joint De-Briefing Session** (Parent and faculty mentors and trainees)

**Trainee Pre-Briefing Session**

Structure: Trainees, faculty and parent mentors in large room setting

Trainees: Arrive and are seated in their pre-organized IPE Teams

Procedure: See sample schedule below

**Table:**

Time	Focus
12:50–1:00	Trainees arrive and are seated with their IPE Team Members
1:00–1:05	Welcome
1:05–1:15	Introduction to Home-visiting video & video presentation
1:15–1:35	IPE Team Activity; Exploring Medical Home
1:35–1:45	Workshop Instructions

- (Parent mentor facilitates) Welcome, viewing of 10-min Home Visiting Documentary video and introduction to purpose of workshop
- (Faculty mentor facilitates) Procedures and expectations of workshop experience

**Standardized Patient/Parent Training Session**

Structure: Parent mentors in small room setting

Procedure:

**Table:**

Time Allotted Lead Facilitator	Content
12:30	Welcome: (Coordinating team member)
**12:30–1:15pm**	Brief history of Project DOCC (Delivery of Chronic Care) Began in 1994 by three mothers from New York National curriculum, designed by parents of children who have chronic illness or disability, using parents as the teachers. The goal is to enhance the education of medical residents Included Home Visit and Parent Mentor Interview Begin PowerPoint Training Program
**1:15pm**	Ground Rules Do not verbally attack any particular medical facility or medical staff The learners are encouraged to ask additional questions, not just those included on the Chronic Illness History. If parent is uncomfortable with any question, parent can respond by saying, ‘I’d prefer not to answer that question’ Stay focused on the question asked and avoid going ‘adrift’. This will ensure that the learners are able to meet the objectives of the workshop experience If parent feels uncomfortable, parent may end the interview at any time. Simply ask to end. Those of us in the observation room will assist parent Soap-box items need to only be shared when parent is given the 5-min warning ‘Knock’ towards the end of the interview time period
**1:20–1:45**	Review Soap-box items: *It should be something that involves policies and procedures for any/all persons with special needs and not an issue that just affects one parent’s situation. An example might be: ‘School nurses should be mandated in every school’* Participating parents share their soap-box topic. Parent and faculty mentors will assist in making any necessary improvements to the items
**1:45**	Workshop Interviews

**Workshop Experience/Parent Mentor Interview**

**Parent Mentor Interview/Exam Room Experience**

Structure: IPE team of trainees and parent mentor in workshop exam room setting

Facilitator: Identify trainee who will play the role of Primary Care Provider and facilitate the conversations between the team and parent

Time Allotted: 40 minutes + 5 minutes for Soap Box Issue (Total = 45 min)

Procedure:

- As a multi-disciplinary team trainees will ask questions of the parent mentor to assess the family’s experience with care and to what degree that care was patient-family-centered.

End of Experience: Learners return to Briefing Room; Parent mentors complete survey and return to a separate room.

**Parent Mentor Interview/Observation Area**

Faculty, Evaluators, and other staff will be observing the exam rooms ‘live’ in a remote location of the workshop lab.

**De-Briefing Session**

**Table:**

**2:30–3:00 Trainees** Return to Briefing room to process with faculty mentors	**2:30–3:00 PARENT Mentors** Return to separate room to process with coordinating team member Complete Survey
**3:00 BOTH Trainees AND Parent mentors** in Briefing Room; Facilitated by faculty mentor	

Faculty Debriefing Session:

**Feedback Questions to ask Trainees**:

- How does your discipline play a role in this parent’s experience?
- What are the challenges of implementing the Patient-Family-Centered Medical Home model for these more complex pediatric patients?
- How does idea of placing the parent in a pivotal ‘teaching’ role, address some of these challenges?
- How might this change the way you practice?
- How might this change outcomes for parents and CSHCN?

**MEASURES**

Trainees will complete a pre and post assessment

Parent mentors complete a survey immediately after the workshop experience

## Appendix B Appendix B

**Available at**: **http://pediatrics.aappublications.org/cgi/pmidlookup?view=long&pmid=22291118**

### Appendix C

**People First Language**

**What do you call a person with a disability? *A person*.**

What words define who you are? The color of your skin or hair? Your age? Your weight? Of course not.

When words alone define a person, the result is a label – a label that often reinforces barriers cre ated by negative and stereotypical attitudes. Every individual deserves to be treated with dignity and respect – regardless of gender, ethnicity, religion, sexual orientation, hair color, or anything else.

People First Language

People First Language is an objective and respectful way to speak about people with disabilities by emphasizing the person first, rather than the disability. It acknowledges what a person *has*, and recognizes that a person *is not* the disability. In putting the person before the disability, People First Language highlights a person’s value, individuality and capabilities.

What should you say?

When referring to individuals with disabilities, be considerate when choosing your words. Focus on the person – and never use terms that label, generalize, stereotype, devalue or discriminate. Unless it is relevant to the conversation, you do not even need to refer to or mention the disability.

The following chart has some examples of People First Language.

**Table:**

Say This	Not This
people with disabilities	the handicapped, the disabled
people without disabilities	normal, healthy, whole or typical people
person who has a congenital disability	person with a birth defect
person who has (or has been diagnosed with) …	person afflicted with, suffers from, a victim of …
person who has Down syndrome	Downs person, mongoloid, mongol
person who has (or has been diagnosed with) autism	the autistic
person with quadriplegia, person with paraplegia, person diagnosed with a physical disability	a quadriplegic, a paraplegic
person with a physical disability	a cripple
person of short stature, little person	a dwarf, a midget
person who is unable to speak, person who uses a communication device	dumb, mute
people who are blind, person who is visually impaired	the blind
person with a learning disability	learning disabled
person diagnosed with a mental health condition	crazy, insane, psycho, mentally ill, emotionally disturbed, demented
person diagnosed with a cognitive disability or with an intellectual and developmental disability	mentally retarded, retarded, slow, idiot, moron
student who receives special education services	special ed student, special education student
person who uses a wheelchair or a mobility chair	confined to a wheelchair; wheelchair bound
accessible parking, bathrooms, etc.	handicapped parking, bathrooms, etc.

### Idioma de las Personas Primero

**¿Cómo se le llama a una persona con una discapacidad? *Una persona*.**

¿Cuáles son las palabras que lo de- finen a usted cómo es? ¿El color de su piel o de su cabello? ¿Su edad?

¿Su peso? Claro que no.

Cuando sólo se usan palabras para definir a una persona, el resul- tado es una etiqueta (una etiqueta refuerza las barreas que se crean por actitudes negativas y estereo- típicas). Cada individuo merece ser tratado con dignidad y respecto, sin importar su sexo, origen étnico, religión, orientación sexual, color de su cabello, o ninguna otra cosa.

**Idioma de las Personas Primero** El Idioma de las Personas Primero es una forma objetiva y respetuosa de hablar acerca de las personas con discapacidades al hacer énfasis en la persona primero, en vez de

su discapacidad. Reconoce lo que la persona tiene, y reconoce que una persona no es la discapacidad. Al poner a la persona antes de la discapacidad, el Idioma de las Per- sonas Primero destaca el valor, la individualidad y las capacidades de una persona.

¿Qué debe decir?

Cuando se dirige a individuos con discapacidades sea cuidadoso con las palabras que selecciona. En- fóquese en la persona (y nunca use palabras que etiquetan, generali- zan, encasillan o discriminan). No necesita hacer referencia o mencio- nar la discapacidad, salvo que sea relevante para la conversación.

La siguiente tabla muestra al- gunos ejemplos del Idioma de las Personas Primero.

**Table:**

Diga esto	No esto
personas con discapacidades	los discapacitados, los inválidos
personas sin discapacidades	personas normales, sanas, enteras o típicas
personas que tienen una discapacidad congénita	personas con un defecto de nacimiento
persona que tiene (o ha sido diagnosticada con) …	persona aquejada con, sufre de, una víctima de …
persona que tiene síndrome de Down	persona Down, mongólico
persona que tiene (o ha sido diagnosticada con) autismo	el autista
persona con tetraplejia, persona con paraplejia, persona diagnosticada con una discapacidad física	una tetrapléjico, un parapléjico
persona con una discapacidad física	un tullido
persona de estatura corta, persona pequeña	un enano
persona incapaz de hablar, persona que usa un dispositivo de comunicación	tonto, mudo
personas que están ciegas, personas con problemas de la vista	los ciegos
persona con una discapacidad del aprendizaje	discapacitado en el aprendizaje
persona diagnosticada con una condición de salud men- tal	loco, psicópata, enfermo mental, trastornado emocional, demente
persona diagnosticada con una discapacidad cognitiva o con una discapacidad intelectual o en el desarrollo	retardado mental, retardado, lento, idiota, tarado
estudiante que recibe servicios educativos especiales	estudiante de educación especial, estudiante ‘special ed’
persona que usa una silla de ruedas o silla para la movilidad	limitado a una silla de ruedas, constreñido a una silla de ruedas
estacionamiento, servicios sanitarios accesibles, etc.	estacionamiento, servicios sanitarios accesibles, etc. para discapacitados

## Appendix D Appendix D. Project DOCC- Team Activity

DISCUSSION SHEET

Less than 55% of families of children with special healthcare needs experience patient-centered care within a medical home

*Please download and have this available for discussion on simulation day.*

**Team Task #1**: Discuss the following:

- What is a medical home? How should it function?
- What is patient-centered care?
- What barriers to receiving patient-centered care within a medical home do families experience?

**Team Task #2:**

Using the seven qualities that specify a medical home, think of a question related to each category to ask the parents during the simulation interview.

**Table:**

**Accessible**	Patients are able to access services with shorter waiting times, ‘after hours’ care, 24/7 electronic or telephone access, and strong communication through health IT innovations.
**Patient/Family-centered**	A partnership among practitioners, patients, and their families ensures that decisions respect patients’ wants, needs, and preferences, and that patients have the education and support they need to make decisions and participate in their own care.
**Continuous**	Eliminate or substantially reduce insurance coverage gaps. Ensure continuous access and use
**Comprehensive**	A team of care providers is wholly accountable for a patient’s physical and mental health care needs, including prevention and wellness, acute care, and chronic care.
**Coordinated**	Care is organized across all elements of the broader health care system, including specialty care, hospitals, home health care, community services and supports.
**Compassionate**	physician should look for signs of trauma and screen for social determinants of health, such as access to nutritious food, safe housing and education.
**Culturally Effective**	equitable health care that is sensitive to cultural difference, mindful of global health concerns and supports resilience and integration into the community

## Appendix E Appendix E. Parent Mentor Preparation Materials

**Notification of Participation Letter (Sample)**

Date

Dear ________________ (Parent),

Thank you for agreeing to participate in the [your institution] Family Experience/Project DOCC (Delivery of Chronic Care) simulation on date. By joining our team, you will forever impact the medical care of children with special health-care needs and their families. The experience that you are creating for future practitioners will positively affect their practices for years to come.

You should have recently received a packet from us containing financial documents needed to reimburse you for your participation in the simulation. You can either mail those forms back to us or bring them with you on the day of the simulation.

In this letter, you will find information and instructions that will assist you in preparing for this event. Enclosed are the following:

- Introduction Index Card
- Soap-box Card
- Instructions regarding the completion of the above-mentioned cards
- Directions to the WVU STEPS Center and parking
- Simulation day schedule
- Additional instructions

Please complete the Introduction and Soap-box cards and bring them with you on the day of the simulation. Please feel free to contact me with any additional questions or concerns. We look forward to having you share your story and be a part of the Family Experience/Project DOCC.

Sincerely,

***If you have an emergency and are not able to attend on the day of the simulation please notify __________________.***

**Introduction Index Card**: Please complete the enclosed card and bring it with you the day of the simulation. Introductions are essential. The magic of Project DOCC’s success is that we speak for each other as parents of children with special health-care needs. Our personal experiences are the basis of this simulation’s curriculum. We are spokespeople for our entire population of families caring for a person with special needs, so we start out by listening to each person’s introduction of themselves and their life experiences.

**Soap-Box Card**: Please complete the enclosed card and bring it with you the day of simulation.

At the end of the interview session with trainees, a few minutes will be given for you to share a ‘soap-box’ issue with them. This issue should be something that involves policies and procedures for all persons with special needs and not just an issue that effects your situation. An example would be: ‘School nurses should be mandated in every school.’ OR ‘The doctors should talk to parents prior to sharing information with their children’.

**Directions to [location] and Parking:**

**Simulation Day Schedule and Expectations:**

**Time Activity Description**

12:20pmReport to [location]

12:30pmParent Mentor Training

1:30pmSimulation Experience/Parent Mentor Interviews

2:30pmDebriefing Session

3:30pmComplete Evaluation prior to leaving

Please dress respectfully. Choose comfortable, clean clothing. Avoid wearing sweats or pajamas. You may bring a spouse, family member or friend. Please let us know ahead of time so that proper arrangements can be made for your guest. Light refreshments (coffee, water, snacks) will be available during the Parent Mentor Training. No food or drink will be permitted during the Parent Mentor Interview.

## Appendix F

**Appendix F. Introduction Card and Soap-Box**

**Introduction Card**

Please you the following format when introducing yourself during the Family Experience Simulation.

**Table:**

Welcome! I am _________________. I have _______ children. Describe your family: ________________________________________ __________________________________________________________ Name of child: _______________ Age: _____ Gender: _____ Diagnoses: ________________________________________________ __________________________________________________________ Basic care needs & medical equipment used: _____________________ __________________________________________________________ __________________________________________________________ School participation: _________________________________________ __________________________________________________________ Explain why you chose to be involved in the simulation: _____________ __________________________________________________________

Toward the end of the simulation interview, a few minutes will be given for you to share one ‘soap-box’ topic of deep concern that involves policies and procedures regarding children with special health-care needs. This issue should be something that broadly affects many families.

## Appendix G

**Soap-box Issue**

**Appendix G. Parent Mentor Preparation Materials**

**Sample: Parent Mentor Interview Questions**

Dear Participating Parent Mentor,

This list is provided to give a general idea of the types of questions you may be asked during the simulation experience. The trainees in this simulation will not have a list of questions in hand but will instead guide you into conversations that will help them understand your experience within the medical home and the impact that your child’s chronic illness has had on the daily life of your family. You are free to decline to answer any questions asked of you during the simulation.

1. Were you offered adequate explanations of tests, diagnoses, treatments, etc. regarding your child?
2. If your first request for explanations or testing was rejected, when did you voice your concerns again and to whom?
3. Were you asked your opinion and given a say regarding your child’s care?
4. Do you feel that your opinions, observations and requests regarding your child’s health were respected by the healthcare provider?
5. Did you feel you were given adequate time to ask questions, voice concerns or request options for future care?
6. When your child’s health status changed were the consequences of new interventions adequately explained to you?
7. Who educated you about the care of your child?
8. Was there a pivotal physician or other healthcare provider who coordinated your child’s health professionals, procedures and information?
9. What qualities did you appreciate most about your child’s healthcare providers?
10. Did you have an opportunity to discuss your child’s medication concerns or questions with a pharmacist?
11. How well were your child’s medications explained to you?
12. Have you had difficulty negotiating your child’s educational needs as related to his/her special healthcare needs?
13. How do you feel the communication has been between you and the members of your medical home?

**Standardized Patient Post Experience Survey (Parents)**

How well did the learner:

**Table:**

	Not at all	Not Very Well	Somewhat	Very Well	Completely
1.Grasp the concept of patient-centered care within a coordinated medical home?	()	()	()	()	()
2. Communicate with you	()	()	()	()	()
3. Develop a working knowledge of the specific medical problems of your child	()	()	()	()	()
4. Show a positive and compassionate attitude toward you and your family	()	()	()	()	()
5. Seek additional encounters and ask questions	()	()	()	()	()
6. Introduce themselves and describe the approach they will use as a team for today’s interview	()	()	()	()	()
7. Show an interest in outside reading and research	()	()	()	()	()
8. Create a comfortable setting	()	()	()	()	()
9. Use people first language	()	()	()	()	()

1. What could have done better/differently?
2. Was the Learner culturally sensitive?
3. How has this training experience impacted you, your ability to advocate for your child, and any future relationships with healthcare providers?

Pre-Encounter Learner

Baseline Pre-Assessment

**Table:**

This is a baseline survey that you must take BEFORE simulation.	
13. On scale of 1–5, with 1 being ‘Not at all aware,’ how aware are you of the concept of Patient Centered Medical Home (PCMH)?	() 1
	() 2
	() 3
	() 4
	() 5
14. List 4 of the essential elements of a patient-centered medical home.	
**On scale of 1–5 with 1 being ‘Not at all aware’, how well do you understand the role your future profession plays in providing care to children with special health care needs regarding Patient Centered Medical Home?**	
15. Comprehensive & Continuous Care	() 1
	() 2
	() 3
	() 4
	() 5
16. Patient, Family-Centered Partnership	() 1
	() 2
	() 3
	() 4
	() 5
17. Planned Coordinated Care	() 1
	() 2
	() 3
	() 4
	() 5
18. Accessible Services	() 1
	() 2
	() 3
	() 4

**Table:**

	() 5
19. Culturally Effective Communication	() 1
	() 2
	() 3
	() 4
	() 5
20. Which of the following clinical risk groups constitutes a medically complex pediatric patient (check all that apply):	[] Healthy- No chronic health problems
	[] History of significant acute
	[] Single minor chronic disease
	[] Minor chronic disease in
	[] Single dominant or moderate
	[] Significant chronic disease in
	[] Significant chronic disease in 3
	[] Dominant and metastatic
	[] Catastrophic condition status

**Table:**

21. On a scale of 1–5 with 1 being ‘No experience,’ how much experience have you had participating in the care of a patient who is medically complex?	() 1
	() 2
	() 3
	() 4
	() 5
22. On a scale of 1–5 with 1 being ‘Not at all’, what knowledge do you have of the challenges families face in caring for their medically complex children?	() 1
	() 2
	() 3
	() 4
	() 5

**Table:**

23. List the essential team members in a PCMH model.
24. Identify some common barriers to successful implementation of the patient-centered medical home model.

Post-Encounter Learner

Post Assessment

Revised 8.26.2019

**Table:**

***This survey is to be filled out **AFTER** your simulation. If you have not yet participated in your simulation, please click the **PRE-ENCOUNTER LEARNER PART** link above and complete that surveyinstead.***	
25. On scale of 1–5, with 1 being ‘Not at all aware,’ how aware are you of the concept of Patient Centered Medical Home (PCMH)?	() 1
	() 2
	() 3
	() 4
	() 5
26. List 4 of the essential elements of a patient-centered medical home.	
On scale of 1–5 with 1 being ‘Not at all aware’, how well do you understand the role your future profession plays in providing care to children with special health care needs regarding Patient Centered Medical Home?	
27. Comprehensive & Continuous Care	() 1
	() 2
	() 3
	() 4
	() 5
28. Patient, Family-Centered Partnership	() 1

**Table:**

	() 2
	() 3
	() 4
	() 5
29. Planned Coordinated Care	() 1
	() 2
	() 3
	() 4
	() 5
30. Accessible Services	() 1
	() 2
	() 3
	() 4
	() 5
31. Culturally Effective Communication	() 1
	() 2
	() 3
	() 4
	() 5

**Table:**

32. Which of the following clinical risk groups constitutes a medically complex pediatric patient (check all that apply):	[] Healthy- No chronic health problems
	[] History of significant acute disease
	[] Single minor chronic disease
	[] Minor chronic disease in multiple organ systems
	[] Single dominant or moderate chronic
	[] Significant chronic disease in multiple organ systems (pairs)
	[] Significant chronic disease in 3 or more organ
	[] Dominant and metastatic malignancies
	[] Catastrophic condition status
33. On a scale of 1–5 with 1 being ‘Not at all’, what knowledge do you have of the challenges families face in caring for their medically complex children?	() 1
	() 2
	() 3
	() 4
	() 5
34. List the essential team members in a PCMH model.	
35. Identify some common barriers to successful implementation of the patient-centered medical home model.	
On a scale of 1 (not at all) to 5 (to a great extent) please rate the following aspects of this simulation exercise in terms of their impact on your understanding of the medical home model and approach to children with special health care needs and their families:

**Table:**

41. Developed a working knowledge of the specific medical problems of the patient being followed	() 1
	() 2
	() 3
	() 4
	() 5
42. Showed a positive and compassionate attitude toward the participating parent/caregiver.	() 1
	() 2
	() 3
	() 4
	() 5
43. Sought additional encounters and asked questions	() 1
	() 2
	() 3
	() 4
	() 5
44. What improvements would you like to see made to this simulation exercise?	
